# Morel-Lavallée Lesion Following a Low-speed Injury: A Case Report

**DOI:** 10.5811/cpcem.2020.7.48358

**Published:** 2020-09-09

**Authors:** Daniel Porter, Jeff Conley, John Ashurst

**Affiliations:** Kingman Regional Medical Center, Department of Emergency Medicine, Kingman, Arizona

**Keywords:** Morel-Lavallee, trauma, internal degloving

## Abstract

**Introduction:**

Soft tissue injuries are a common presenting complaint seen in the emergency department following trauma. However, internal degloving injuries are not commonly seen by the emergency provider.

**Case Report:**

A 57-year-old male presented with right lower extremity pain, bruising, and swelling after a low-speed bicycle accident five days prior. Physical examination revealed an edematous and ecchymotic right lower extremity extending from the mid-thigh distally. Computed tomography of the thigh demonstrated a hyperdense foci within the fluid collection suggesting internal hemorrhage and internal de-gloving suggestive of a Morel-Lavallée lesion.

**Discussion:**

The Morel-Lavallée lesion is a post-traumatic soft tissue injury that occurs as a result of shearing forces that create a potential space for the collection of blood, lymph, and fat. First described in 1853 by French physician Maurice Morel-Lavallée, this internal degloving injury can serve as a nidus of infection if not treated appropriately. Magnetic resonance imaging has become the diagnostic modality of choice due to its high resolution of soft tissue injuries. Treatment has been focused on either conservative management or surgical debridement after consultation with a surgeon.

**Conclusion:**

The emergency physician should consider Morel-Lavallée lesions in patients with a traumatic hematoma formation to avoid complications that come from delayed diagnosis.

## INTRODUCTION

Trauma is a common presenting complaint to the emergency department (ED) and is the leading cause of morbidity, hospitalizations, and death in Americans 1–45 years old.^1^ Morel-Lavallée lesions are post-traumatic injuries following blunt trauma to soft tissues and often can either go undiagnosed or present weeks after the initial injury.^2^ Typically seen after a high-speed injury, the traumatic shearing mechanism creates a potential space for fluid accumulation by separating the subcutaneous tissue from the underlying deeper fascial layers causing an internal degloving injury.^2^ This newly created space allows for the collection of blood, lymph, and occasionally necrotic fat.^2^ There could be a delay in diagnosis as these lesions often occur in patients with multisystem trauma, which increases the likelihood of infection, tissue necrosis, and pseudocapsule formation.^3^,^4^ We present a case of a Morel-Lavallée lesion following a low-speed bicycle accident that was treated with conservative management.

## CASE REPORT

A 57-year-old male presented to the ED with right lower extremity pain, bruising, and swelling after striking his leg on the sidewalk following a low-speed bicycle accident five days prior. He noted no other injuries from the accident but his leg had increased in size over the preceding several days and began to develop wounds. He stated no past medical history and took no medications on a daily basis.

**Image 1:**
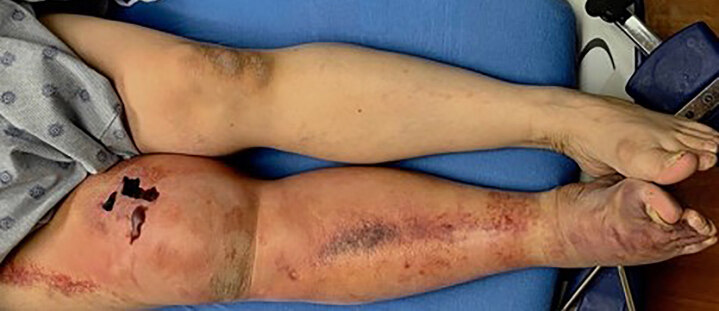
Large area of ecchymosis of the right lower extremity with hemorrhagic bullae formation (arrow).

Physical examination revealed an edematous and ecchymotic right lower extremity extending from the mid-thigh distally (Image 1). There were areas of hemorrhagic bullae along the medial thigh and there was a 1+ dorsalis pedis pulse in the extremity. The remainder of the trauma examination was within normal limits.

**Image 2:**
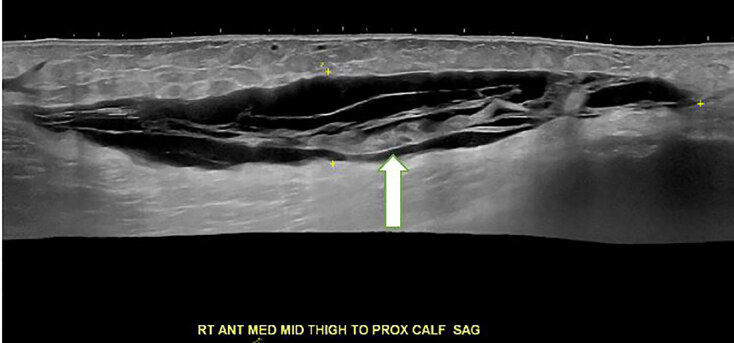
Ultrasound of the right lower extremity depicting the formation of a hypoechoic area between muscle and fascia (arrow).

**Image 3:**
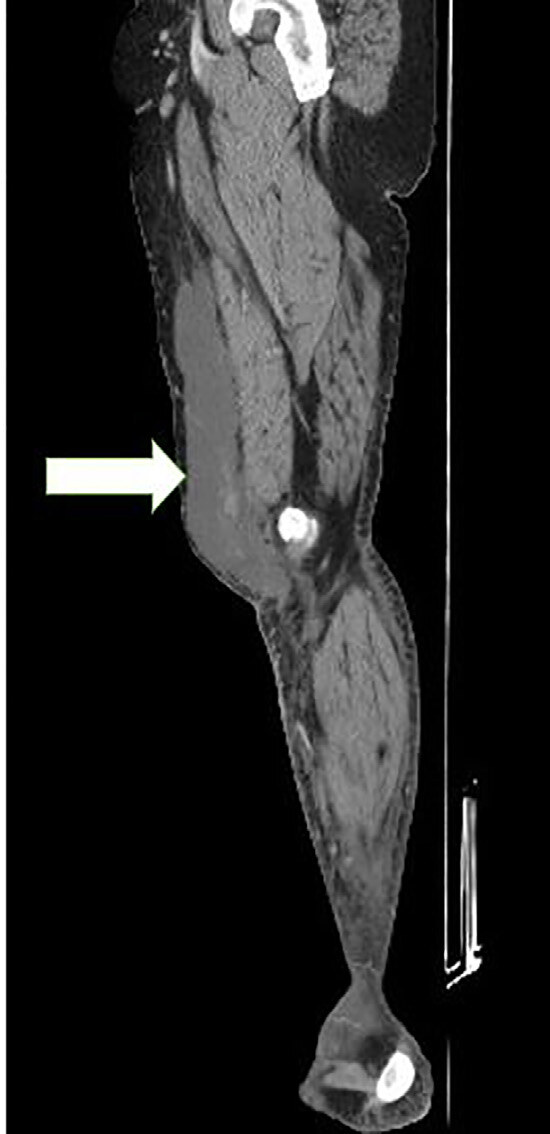
Computed tomography of the right lower extremity showing an internal hemorrhage and internal degloving injury of the thigh (arrow).

Ultrasonography revealed a 20 centimeters (cm) × 4 cm × 14 cm hypoechoic fluid collection in the right medial thigh (Image 2). Computed tomography of the thigh demonstrated a hyperdense foci within the fluid collection suggesting internal hemorrhage and internal degloving injury suggestive of a Morel-Lavallée lesion (Image 3).

CPC-EM Capsule

What do we already know about this clinical entity?

*A Morel-Lavallée lesion is a post-traumatic soft tissue injury that creates a potential space for the collection of blood, lymph, and fat*.

What makes this presentation of disease reportable?

*While typically presenting after a high-speed injury, we present this case that occurred following a low-speed bicycle accident*.

What is the major learning point?

*Emergency department management should include consultation with a surgeon to determine best treatment strategies*.

How might this improve emergency medicine practice?

*Providers should be vigilant in their examination of all injured areas, obtain diagnostics studies, and surgical consultation to prevent long-term complications*.

The patient underwent bedside needle drainage of the fluid collection by general surgery, which yielded over 500 milliliters of sanguineous fluid. He was discharged with a compression dressing and general surgery follow-up. The patient unfortunately did not follow up with general surgery as an outpatient and was seen in the ED several weeks later with a deep soft tissue infection of the thigh requiring surgical debridement.

## DISCUSSION

Typically occurring after direct trauma to the pelvis, thigh, or knee, a Morel-Lavallée lesion is caused by a shearing force to an area with strong underlying fascia.^5^ Following disruption of the fascial layer, transaponeurotic capillaries and lymphatic vessels become disrupted and leak haemolymphatic fluids into the newly formed cavity.^6^ Over time, blood is reabsorbed and replaced with serosanguinous fluids, which can cause a sustained inflammatory response and the formation of a cystic mass.^5^

Although the exact epidemiology is unknown, the majority of Morel-Lavallée injuries are seen in those with a body mass index of 25 or more following trauma with a high-energy mechanism.^5^,^6^ Upon initial ED presentation, a patient may have soft tissue swelling with or without ecchymosis, skin contour asymmetry with hypermobility, or a soft tissue fluctuance with minimal or no tenderness.^5^,^6^ Given many of these injuries do not become apparent until after the initial injury, patients may also present in a delayed fashion with decreased sensation, necrosis, and color changes over the lesion.^5^,^6^

Magnetic resonance imaging is currently the imaging modality of choice due to its high-contrast resolution, multiplanar image acquisition, and enhanced anatomical details as compared to other modalities.^2^ Computed tomography is another diagnostic modality that can be used because of its availability in the trauma setting but does not easily allow for the characterization of soft tissue injuries.^2^ Ultrasonography has also been used in the diagnosis of Morel-Lavallée lesions because of its low cost and the allowance for dynamic imaging.^2^ However, ultrasonography is highly operator dependent and cannot be performed on areas with open wounds.^2^

Once diagnosed, ED management should include consultation with a surgeon to determine best treatment strategies to prevent long-term sequelae. Currently no validated treatment algorithm exists for Morel-Lavallée lesions and treatment has been based upon surgeon preference, lesion size, and stage of the injury.^5^ Conservative management can include compression dressings or aspiration of fluid, while surgical options include open debridement, limited incision with drainage, and sclerodesis of the injury.^5^ Complications from the injury or procedure can present at any time during the patient’s course and can include recurrence of the lesion, infection, necrosis, and contour deformities of the affected area.^5^

## CONCLUSION

Morel-Lavallée lesions represent a rare but serious traumatic injury that could lead to devastating long-term morbidity. The emergency provider should be vigilant in his or her examination of all injured areas and obtain diagnostics studies coupled with surgical consultation to prevent long-term complications.
